# Palbociclib induces activation of AMPK and inhibits hepatocellular carcinoma in a CDK4/6‐independent manner

**DOI:** 10.1002/1878-0261.12072

**Published:** 2017-05-15

**Authors:** Feng‐Shu Hsieh, Yao‐Li Chen, Man‐Hsin Hung, Pei‐Yi Chu, Ming‐Hsien Tsai, Li‐Ju Chen, Yung‐Jen Hsiao, Chih‐Ting Shih, Mao‐Ju Chang, Tzu‐I Chao, Chung‐Wai Shiau, Kuen‐Feng Chen

**Affiliations:** ^1^ Department of Medical Research National Taiwan University Hospital Taipei Taiwan; ^2^ National Center of Excellence for Clinical Trial and Research National Taiwan University Hospital Taipei Taiwan; ^3^ Department of Surgery Changhua Christian Hospital Taiwan; ^4^ School of Medicine Kaohsiung Medical University Taiwan; ^5^ Division of Medical Oncology Department of Oncology Taipei Veterans General Hospital Taiwan; ^6^ School of Medicine National Yang‐Ming University Taipei Taiwan; ^7^ Department of Pathology Show Chwan Memorial Hospital Changhua Taiwan; ^8^ School of Medicine College of Medicine Fu‐Jen Catholic University New Taipei Taiwan; ^9^ Transplant Medicine & Surgery Research Centre Changhua Christian Hospital Taiwan; ^10^ Institute of Biopharmaceutical Sciences National Yang‐Ming University Taipei Taiwan

**Keywords:** abemaciclib, AMPK, HCC, palbociclib, PP5, ribociclib

## Abstract

Palbociclib, a CDK4/6 inhibitor, has recently been approved for hormone receptor‐positive breast cancer patients. The effects of palbociclib as a treatment for other malignancies, including hepatocellular carcinoma (HCC), are of great clinical interest and are under active investigation. Here, we report the effects and a novel mechanism of action of palbociclib in HCC. We found that palbociclib induced both autophagy and apoptosis in HCC cells through a mechanism involving 5′ AMP‐activated protein kinase (AMPK) activation and protein phosphatase 5 (PP5) inhibition. Blockade of AMPK signals or ectopic expression of PP5 counteracted the effect of palbociclib, confirming the involvement of the PP5/AMPK axis in palbociclib‐mediated HCC cell death. However, CDK4/6 inhibition by lentivirus‐mediated shRNA expression did not reproduce the effect of palbociclib‐treated cells, suggesting that the anti‐HCC effect of palbociclib is independent of CDK4/6. Moreover, two other CDK4/6 inhibitors (ribociclib and abemaciclib) had minimal effects on HCC cell viability and the PP5/AMPK axis. Palbociclib also demonstrated significant tumor‐suppressive activity in a HCC xenograft model, which was associated with upregulation of pAMPK and PP5 inhibition. Finally, we analyzed 153 HCC clinical samples and found that PP5 expression was highly tumor specific and was associated with poor clinical features. Taken together, we conclude that palbociclib exerted antitumor activity against HCC through the PP5/AMPK axis independent of CDK4/6. Our findings provide a novel mechanistic basis for palbociclib and reveal the therapeutic potential of targeting PP5/AMPK signaling with a PP5 inhibitor for the treatment of hepatocellular carcinoma.

**Abbreviations**
3‐MA: 3‐methyladenine
AA: arachidonic acid
AMPK: 5′ AMP‐activated protein kinase
HCC: hepatocellular carcinoma
IHC: immunohistochemistry
IP: immunoprecipitation
PARP: poly (ADP‐ribose) polymerase
PD: palbociclib
PP5: protein phosphatase 5
SC: subcutaneous(ly)

## 1 Introduction

Over the past decade, the incidence of hepatocellular carcinoma (HCC) has drastically increased. Of particular note, in the Asia‐Pacific region, there are 500 000 new cases every year (Lin and Kao, 2013). Surgical treatment is the most effective way to treat liver cancer. However, a low curative resection and high recurrent metastasis ratio compromise the treatment efficacy (Matsuda and Saika, 2012; Sintra *et al*., 2013). Furthermore, there are limited treatment options for patients. So far, the multikinase inhibitor, sorafenib (Nexavar), is the only approved targeted drug for patients with advanced HCC. It is challenging to develop effective therapies for HCC treatment.

The field of cell cycle inhibitors has been revitalized due to the recent clinical success of palbociclib (PD‐0332991; IBRANCE), a potent and highly selective CDK4/6 inhibitor developed by Pfizer (New York, NY, USA). Palbociclib plus letrozole significantly improved the progression‐free survival (20.2 months for palbociclib plus letrozole vs. 10.2 months for letrozole alone) in women with advanced estrogen receptor‐positive and HER2‐negative breast cancer (Finn *et al*., 2015). Based on the trial outcome, the United States Food and Drug Administration (FDA) approved palbociclib for advanced breast cancer treatment in February 2015. In addition to breast cancer, palbociclib has been found to inhibit cell growth and exert antitumor activity in various preclinical cancer models (Baughn *et al*., 2006; Fry *et al*., 2004; Konecny *et al*., 2011; Michaud *et al*., 2010). For the treatment of liver cancer, an ongoing phase II trial is testing the effects of palbociclib in patients who failed or show intolerance to sorafenib (NCT01356628).

There is growing awareness that activation of AMP‐activated protein kinase (AMPK) may oppose tumor progression in various cancer types, including liver cancer (Li *et al*., 2015). In HCC patient samples, decreased AMPK activity was associated with poor prognosis and aggressive clinicopathologic features (Zheng *et al*., 2013). Pharmacologically raised AMPK activity inhibited growth of HCC cells and xenograft tumors by inducing senescence, autophagy, and apoptosis (Cheng *et al*., 2014; Hu *et al*., 2014; Yi *et al*., 2013; Yu *et al*., 2014). These findings suggest that AMPK is emerging as a promising target for HCC treatment. It has been generally believed that the CDK4/6‐RB/E2F axis mediates the molecular activity of palbociclib (Asghar *et al*., 2015); however, recently, the antiproliferative effect of palbociclib in HCC cells has been examined, and interestingly, palbociclib showed activity irrespective of RB status in HCC cells and tumor xenograft (Rivadeneira *et al*., 2010). Here, we further investigated the underlying molecular events associated with RB‐independent anti‐HCC function of palbociclib by focusing on AMPK signaling.

In this report, we explored the anti‐HCC activity of palbociclib *in vivo* and *in vitro*. Additionally, we addressed the mechanism of action of palbociclib in HCC. Our results showed that palbociclib inhibits protein phosphatase 5 (PP5) phosphatase activity that is associated with upregulation of AMPK phosphorylation and increased cytotoxicity in HCC cells. The PP5/AMPK axis but not the CDK4‐RB pathway mediates the cytotoxic effects of palbociclib. Our findings provide mechanistic insights for therapeutic application of palbociclib in HCC‐targeted therapy.

## 2 Materials and methods

### 2.1 Chemicals

For *in vitro* studies, palbociclib (PD‐0332991) was from MedKoo Biosciences (Morrisville, NC, USA); 3‐methyladenine (3‐MA) was from Cayman Chemical (Ann Arbor, MI, USA); arachidonic acid (AA) was from BioVision (Milpitas, CA, USA); ribociclib (LEE011), abemaciclib (LY2835219), compound C (dorsomorphin dihydrochloride), Z‐VAD‐FMK, and metformin were from MedChem Express (Monmouth Junction, NJ, USA). Palbociclib (Cat. No. HY‐50767A) purchased from MedChem Express was used for *in vivo* testing.

### 2.2 Antibodies

PP5 antibody (C‐20) (sc‐32588) was used for immunoblotting, and the PP5 antibody (H‐7) (sc‐271816) used for immunoprecipitation was obtained from Santa Cruz Biotechnology (San Diego, CA, USA). Purified mouse anti‐PP5 antibody (611020) for the immunohistochemistry experiments was from BD Transduction Laboratories (San Jose, CA, USA). Poly (ADP‐ribose) polymerase (PARP) antibody (sc‐8007) was purchased from Santa Cruz Biotechnology. Phospho‐Rb (Ser807/811) (no. 8516), Rb (no. 9309), CDK4 (no. 12790), CDK6 (no. 13331), AMPKα (no. 2532/no. 2757), phospho‐AMPKα (Thr172) (no. 2535), phospho‐ULK1 (Ser317) (no. 12753), ULK1 (no. 8054), phospho‐SAPK/JNK (no. 4668), SAPK/JNK (no. 9252), phospho‐ASK1 (Thr845)(no. 3765), ASK1 (no. 8662), caspase 9 (no. 9502), and LC3B (no. 3868) were obtained from Cell Signaling (Danvers, MA, USA). GAPDH antibody was purchased from Abcam (Cambridge, MA, USA). Mouse monoclonal anti‐DDK (TA50011) and anti‐beta actin (66009‐1) antibodies were from OriGene Technologies, Inc. (Rockville, MD, USA) and ProteinTech (Rosemont, IL, USA), respectively.

### 2.3 Cell culture, transfection, and treatment

Hep3B, PLC5, and Huh7 cells were maintained in Dulbecco's modified Eagle's medium with 10% (v/v) fetal bovine serum. Lipofectamine 2000 reagent (Invitrogen, Carlsbad, CA, USA) was used for transfection with plasmids for transient expression. siRNA transfection was performed using DharmaFECT 4 transfection reagent. For *in vitro* drug treatment, chemicals were dissolved in DMSO or ethanol and added to the cultured cells at the indicated concentration and duration. To inhibit autophagy, HCC cells were preincubated with 1 mm 3‐MA for 2 h before palbociclib treatment.

### 2.4 Immunofluorescence

1 × 10^5^ HCC cells were seeded on coverslips one day before drug treatment. For detecting endogenous LC3 proteins, indirect immunofluorescence was performed. Briefly, cells were fixed in freshly prepared 4% paraformaldehyde for 15 min and permeabilized in PBS containing 0.3% Triton X‐100 for 5 min at room temperature. After blocking in 5% BSA/PBS for 1 h, they were incubated with antibody dilution buffer (1 × PBS/1% BSA/0.3% Triton X‐100) containing anti‐LC3B antibody (Cell Signaling Technology no. 3868; diluted 1 : 400) followed by Alexa Fluor^**®**^ 488‐conjugated secondary antibodies (Jackson ImmunoResearch, West Grove, PA, USA; no. 111‐545‐045, diluted 1 : 400) and DAPI‐Fluoromount‐G^**®**^ (0100‐20; Southern Biotech, Birmingham, AL, USA) stain. Images were analyzed using a fluorescence microscope.

### 2.5 Expression plasmids and RNAi

Human PP5 cDNA containing amino acid 1–499 amplified by PCR was subcloned into pCMV‐tag 2B (Agilent Technologies, Santa Clara, CA, USA) and pGEX‐4T‐1 (GE Healthcare Bio‐sciences, Pittsburgh, PA, USA) to express DDK‐PP5 and GST‐PP5. ON‐TARGETplus SMARTpool siRNA against AMPKα2 (PRKAA2) and ON‐TARGETplus nontargeting (NT) pool siRNA were purchased from Dharmacon (Chicago, IL, USA). For CDK4/6 knockdown, pLKO.1‐puro vector expressing NT control shRNA (pLKO TRC025) or shRNAs targeting CDK4 (TRCN0000000363) and CDK6 (TRCN0000055435) were used. The short hairpin RNA (shRNA) reagents were obtained from the National Core Facility for Manipulation of Gene Function by RNAi, Academia Sinica, supported by the National Core Facility Program for Biotechnology Grants of MOST, Taiwan.

### 2.6 Cell viability and cytotoxicity assays

For MTT assay and DNA fragmentation detection, HCC cells were seeded the day before drug treatment at a density of 5 × 10^3^~1 × 10^4^ cells/well in 0.1 mL culture medium onto the 96‐well tissue culture plates. The cells were exposed to various concentrations of indicated drug for 24 or 48 h. Cell viability was measured by the 3‐[4,5‐dimethylthiazol‐2‐yl]‐2,5‐diphenyltetrazolium bromide (MTT). DNA fragmentation was assayed by cell death ELISA kit (Roche Life Science, Mannheim, Germany) according to the manufacturer's instructions. For sub‐G1 analysis, 2 × 10^5^ HCC cells were seeded on six‐well plates. After 24 h of drug treatment, cells were harvested by trypsin digestion and fixed in 70% ethanol overnight. The cells were stained with 10 μg·mL^−1^ propidium iodide for half an hour. The percentage of cells in sub‐G1 phase was quantified by flow cytometry.

### 2.7 PP5 phosphatase activity

Drug‐treated HCC cells were harvested and lysed in 1% NP‐40‐containing buffer (50 mm of Tris/HCl [pH 8.0], 150 mm of NaCl, 1 mm of PMSF, 1% NP‐40, and 2 mm of EDTA). The cell lysates were precleared with protein G beads (GE Healthcare Bio‐sciences) for at least 2 h. The precleared cell lysates were incubated with anti‐PP5 antibody. After overnight incubation, protein G was added to each sample and the tubes were incubated for another 3 h with rotation at 4 °C. The beads were washed four times with ice‐cold lysis buffer. The tubes were centrifuged at maximum speed after the final wash. The PP5‐containing lysates were then subjected to RediPlate 96 EnzChek Serine/Threonine Phosphatase Assay Kit (R‐33700) for cellular PP5 activity detection (Molecular Probes, Inc., Eugene, OR, USA). For *in vitro* PP5 activity assay, recombinant GST‐PP5 proteins purified from *Escherichia coli* BL21 were used.

### 2.8 Patient samples and immunohistochemical staining

This study was approved by the ethics committee of the Institutional Review Board of Changhua Christian Hospital. All the samples used in this study were obtained from patients with signed informed consent in accordance with the Declaration of Helsinki. In total, 153 primary HCC tumor tissues and a paired normal tissue were obtained from patients receiving surgical resection in Changhua Christian Hospital. All these samples were carefully collected and used a tissue microarray. The slides containing all these tissues were analyzed for the expression of PP5 and quantified by *H*‐score, which took the percentage and the strength of the positively stained cells into consideration.

### 2.9 Xenograft tumor growth

The following experimental procedures were performed in accordance with protocols approved by the Institutional Laboratory Animal Care and Use Committee of National Taiwan University. Five‐week‐old male NCr athymic nude mice were subcutaneously inoculated with 5 × 10^6^ Huh7 cells suspended in 100 μL PBS containing 50% Matrigel. Mice were randomized into vehicle and palbociclib treatment when tumors reached 100–200 mm^3^. Palbociclib was dissolved in lactic acid solution (50 mm, pH 4.0) to a concentration at 15 mg·mL^−1^ and was given orally every three days. The volume of administration was 200 μL. The tumor sizes were measured twice weekly. The tumor weights, PP5 activity, and signal transduction were measured after tumor excision.

### 2.10 Statistical analysis

Data analysis was performed by prism 5 software (GraphPad Software, La Jolla, CA, USA), and Student's *t*‐test was used for pairwise comparisons. For the clinical part, high PP5 expression was defined by *H*‐score higher than 200. Clinical characteristics among patients with high and low PP5 expressions were compared by chi‐square test. All the statistical examinations were conducted by spss software for windows (17.0 version; SPSS, Chicago, IL, USA). *P* values of less than 0.05 were considered significant.

## 3 Results

### 3.1 Palbociclib induces autophagy and apoptosis in hepatocellular carcinoma cells

**Figure 1:**
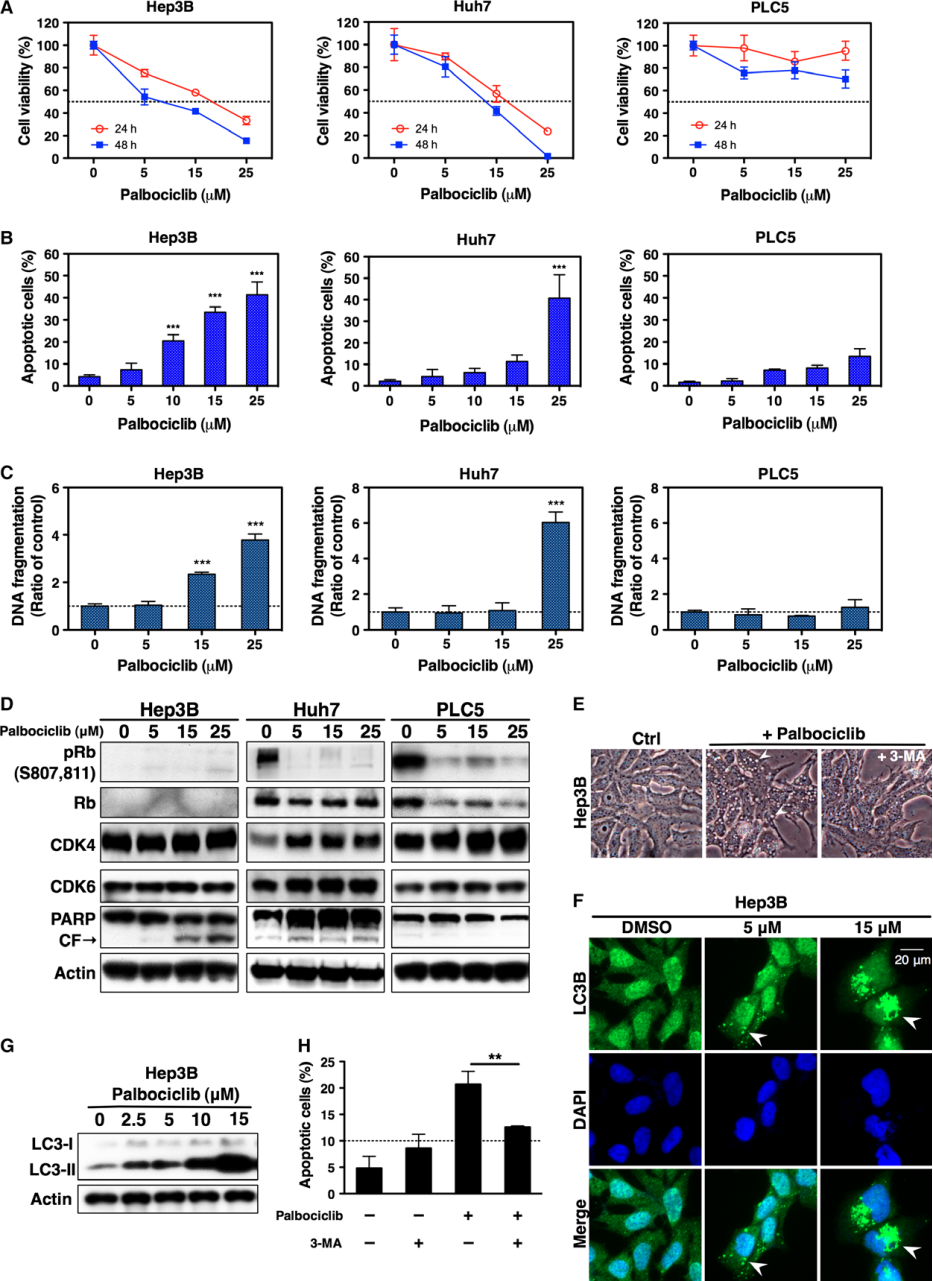
Palbociclib induces autophagy and apoptosis in HCC. (A) Dose‐ and time‐dependent effects of palbociclib on cell viability in three HCC cell lines. Hep3B, Huh7, and PLC5 cells were treated with palbociclib at the indicated concentrations for 24 or 48 h and assayed by MTT. The solvent (DMSO) concentration in each sample is equal (0.7%). (B) Dose escalation effects of palbociclib on sub‐G1 population. After 24 h of drug treatment, the cells were subjected to flow cytometry analysis. Data are mean ± SD of at least three replicates. ***P <* *0.001. (C) Effects of palbociclib on DNA fragmentation. HCC cells were exposed to palbociclib for 24 h and assayed by Cell Death Detection ELISA. (D) Effects of palbociclib on CDK4/6‐Rb pathway. HCC cells were treated with different doses of palbociclib for 24 h, and then, the cells were subjected to western blot analysis. CF, cleaved fragment. (E) Cytoplasmic vacuole induction in palbociclib‐treated Hep3B cells. Hep3B cells were preincubated with 3‐MA (0.5 m, 2 h) and treated with palbociclib (15 μm) for another 8–16 h. After taking images under phase‐contrast microscopy, the cells were subjected to sub‐G1 analysis in (H). Arrowheads indicate cytoplasmic vacuoles. (F) LC3B immunofluorescence in palbociclib‐treated Hep3B cells. Arrowheads indicate LC3‐positive autophagosomes. Nuclei were counterstained with DAPI (blue). (G) Palbociclib increases LC3‐II amount in a dose‐dependent manner. (H) Pretreatment with 3‐methyladenine (3‐MA) counteracts the effect of palbociclib on Hep3B cell apoptosis. **P <* *0.01.

To explore the anti‐HCC activity of palbociclib, we conducted a series of cell viability and cytotoxicity assays in human HCC cell lines (Hep3B, Huh7, and PLC5). As shown in Fig. 1A, palbociclib exhibited a dose‐ and time‐dependent growth inhibitory effect starting from the concentration of 5 μm in Hep3B and Huh7 cell lines. Sub‐G1 analysis, DNA fragmentation assay, and PARP cleavage further revealed that palbociclib could induce cell apoptosis within 24 h (Fig. 1B–D). Palbociclib also caused caspase activation and the pan‐caspase inhibitor, Z‐VAD‐FMK, attenuated palbociclib‐induced cell viability loss (Fig. S1). PLC5 showed resistance to palbociclib as measured by cell viability, apoptosis (IC_50_ > 25 μm), and caspase 3 immunofluorescence (Fig. 1A–C, the right panels; Fig. S1A). Overall, Hep3B and Huh7 were sensitive, while PLC5 was relatively resistant to the effect of palbociclib.

CDK4/6 phosphorylates retinoblastoma protein (Rb) at serine 807/811, leading to E2F release, thus driving G1‐to‐S transition. We therefore probed the phosphorylation status of Rb as a biomarker for CDK 4/6 inhibition by palbociclib. The HCC cell lines Huh7 and PLC5 carry wild‐type Rb, whereas Hep3B (Rb‐deficient hepatoma cell line) has no Rb protein expression. Interestingly, palbociclib caused a marked reduction in Rb phosphorylation at serine 807/811 and slightly upregulated CDK4 and CDK6 in both sensitive Huh7 and resistant PLC5 cells (Fig. 1D). Moreover, the apoptotic activity of palbociclib was retained in Rb‐negative Hep3B cells (Fig. 1A–D, left panels). These results indicate that the apoptotic effect of palbociclib may not be associated with CDK4/6 inhibition as well as Rb levels, and there is likely an alternative pathway mediating the cytotoxic effect of palbociclib.

In addition to apoptosis, autophagic death also contributes to the effects of palbociclib against HCC. Here, we used Hep3B cell line to investigate the Rb‐independent anti‐HCC activity of palbociclib. We observed that Hep3B cells treated with palbociclib undergo morphological changes by forming cytoplasmic vacuolation (Fig. 1E). The effect of palbociclib on autophagy was confirmed by an increase in LC3 puncta in drug‐treated HCC cells (Figs 1F and S2A) and the formation of acidic vesicular organelles (Fig. S2B). In consistent with the aforementioned findings, treatment of Hep3B cells with palbociclib resulted in the upregulation of autophagic marker, LC3‐II, in a concentration‐dependent manner (Fig. 1G). Moreover, the cytoplasmic vacuoles and apoptotic cells decreased when Hep3B cells were pretreated with an autophagy inhibitor, 3‐methyladenine (3‐MA), suggesting that palbociclib‐induced Hep3B cell death is dependent on autophagy induction (Fig. 1E, far right panel and H). Collectively, our results showed that palbociclib possesses potent activities against HCC by inducing both autophagy and apoptosis.

### 3.2 Activation of AMPK is involved in palbociclib‐induced apoptosis and autophagy in HCC cells

**Figure 2:**
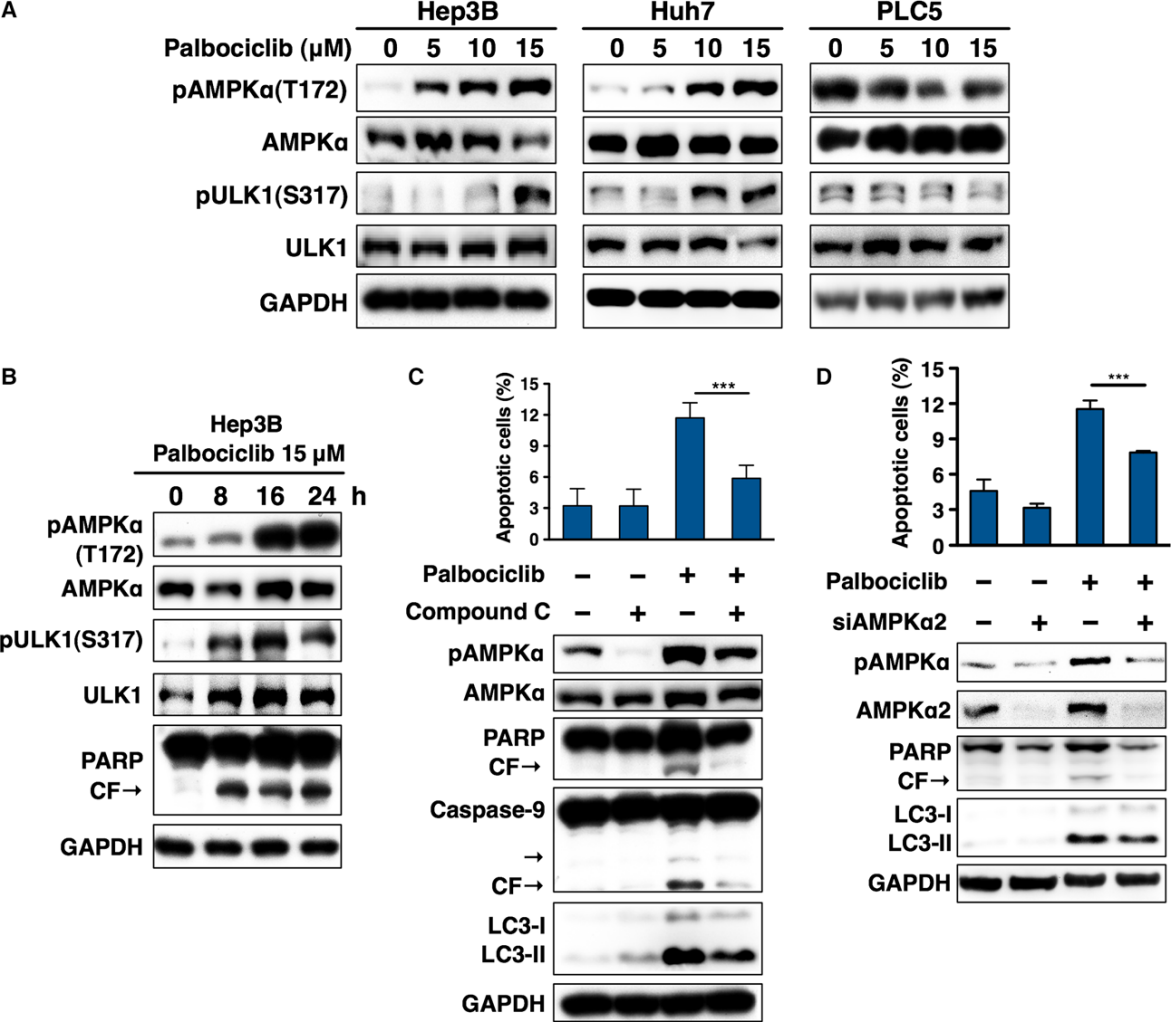
AMPK activation mediates the cytotoxic effects of palbociclib. (A) Dose‐dependent response of palbociclib on AMPK‐related molecules. HCC cells were treated with different concentrations of palbociclib for 24 h. The phosphorylation of the indicated proteins was determined by western blotting. (B) Palbociclib enhances AMPK phosphorylation in a time‐dependent manner. (C) Inhibition of AMPK reverses palbociclib‐induced autophagy and apoptosis. Hep3B cells were incubated with AMPK inhibitor (compound C, 2.5 μm) for 4 h and then treated with palbociclib for 24 h. Apoptotic cells were determined by flow cytometry. (D) Silencing AMPKα by siRNA reduces palbociclib‐triggered cell death. Hep3B cells were transfected with control or AMPKα siRNA for 24 h and treated with palbociclib (15 μm) for another 24 h. Knockdown of AMPKα was confirmed by immunoblotting. ***P < 0.001. CF, cleaved fragment.

To explore the underlying mechanism for the sensitivity of palbociclib, we examined the signal alterations in palbociclib‐treated HCC cells. Interestingly, we found that the phosphorylation status of AMPK at The 172 changed significantly with palbociclib treatment in Hep3B and Huh7 cells (Fig. 2A,B). AMPK is an evolutionarily conserved energy‐sensing protein kinase that is activated under various stresses (Hardie *et al*., 2012; Steinberg and Kemp, 2009). Activation of AMPK requires phosphorylation of its catalytic α subunit on threonine 172. By regulating a variety of downstream targets (mTORC1, p53, ULK1, etc.), AMPK has been implicated as an important regulator for both autophagy and apoptosis (Hoyer‐Hansen and Jaattela, 2007; Shackelford and Shaw, 2009). As shown in Fig. 2A, upregulation of phospho‐AMPK and its downstream phospho‐ULK1 was present in palbociclib‐sensitive Hep3B and Huh7 cells, but not in resistant PLC5 cells, suggesting that activation of AMPK determines the sensitivity of HCC cells to palbociclib. To further verify the importance of AMPK activation, we blocked AMPK signals by AMPK inhibitor, compound C, or siRNA targeting AMPK subunit α2, before palbociclib treatment. As shown in Fig. 2C, palbociclib‐induced apoptosis, PARP and caspase 9 cleavage, and LC3 II conversion were suppressed by AMPK inhibition. Silencing AMPK by siRNA also attenuated the effect of palbociclib on apoptosis and LC3 II conversion, and decreased phospho‐AMPK levels (Fig. 2D). Taken together, these results suggested that AMPK activation mediates the cytotoxic effect of palbociclib in HCC.

### 3.3 Protein phosphatase 5 mediates the effect of palbociclib on AMPK activation, autophagy, and apoptosis

**Figure 3:**
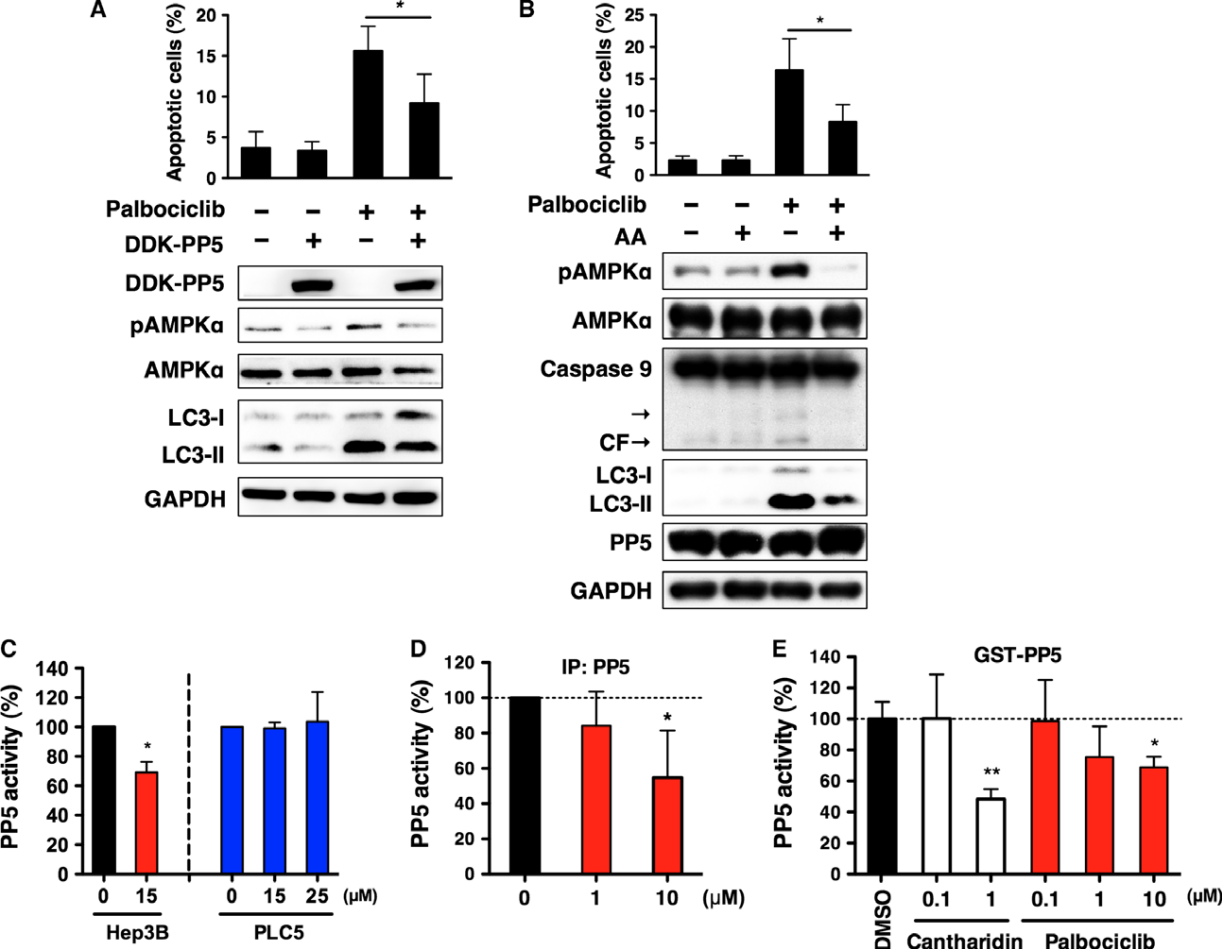
PP5 mediates palbociclib‐induced AMPK activation, autophagy, and apoptosis. (A) PP5 overexpression suppresses the effect of palbociclib on pAMPKα, autophagy, and apoptosis. Hep3B cells were transfected with vector or DDK‐PP5 for 24 h and treated with palbociclib (15 μm) for another 24 h. (B) Pretreatment with arachidonic acid (AA), a PP5 activator, reversed the effect of palbociclib on pAMPKα, autophagy, and apoptosis. Hep3B cells were pretreated with AA (100 μm) for 4 h and then treated with palbociclib for 24 h. Autophagy was determined by LC3 immunoblotting. Apoptotic cells were measured by flow cytometry. *P <* *0.05. (C) PP5 activity in Hep3B and PLC5 cells. (D) Palbociclib inhibits PP5 activity in PP5‐containing Hep3B lysate. (E) Palbociclib suppresses activity of GST‐PP5. Cantharidin, a known Ser/Thr phosphatase inhibitor, served as a positive control. *P <* *0.05

We further investigated how palbociclib, a kinase inhibitor, stimulates AMPK phosphorylation. Intriguingly, we found extensive upregulation of protein serine/threonine phosphorylation levels after palbociclib treatment. The observation implies the involvement of serine/threonine protein phosphatases. We therefore conducted a serine/threonine protein phosphatase screen by examining their substrate proteins and activities. We noticed that palbociclib induced phosphorylation of apoptosis signal‐regulating kinase 1 (ASK1) and c‐Jun N‐terminal kinase (JNK) (Fig. S3), the two members of the MAPK cascade known to be negatively regulated by PP5 (Morita *et al*., 2001; Zhou *et al*., 2004). PP5 has been shown to participate in stress‐induced signaling, such as oxidative stress, DNA damage, and hypoxia (Kang *et al*., 2009; Morita *et al*., 2001; Zhou *et al*., 2004). To see whether PP5 participates in the mechanism of action of palbociclib, the effects of ectopic PP5 or PP5 activator on palbociclib‐induced AMPK activation, autophagy, and apoptosis were determined. Overexpression of PP5 resulted in downregulation of phospho‐AMPK and counteracted the cytotoxic effects of palbociclib (Fig. 3A). Similarly, pretreatment of Hep3B cell with AA, a polyunsaturated fatty acid that stimulates PP5 phosphatase activity (Chen and Cohen, 1997), reduced palbociclib‐mediated AMPK phosphorylation, autophagy, and apoptosis (Fig. 3B). We reasoned that palbociclib stimulates AMPK phosphorylation and exerts its anti‐HCC function through inhibiting PP5 phosphatase activity. To test this hypothesis, we investigated the effects of palbociclib on PP5 phosphatase activity. We found that palbociclib significantly reduced the cellular activity of PP5 in Hep3B cells, but not in the resistant PLC5 cell line (Fig. 3C). Moreover, palbociclib reduced PP5 activity in a dose‐dependent manner when incubated with PP5‐containing cell lysates or purified recombinant PP5 protein *in vitro* (Fig. 3D,E), suggesting that palbociclib may inhibit the activity of PP5 via direct interaction. Cantharidin, a potent serine/threonine protein phosphatase inhibitor, served as a positive control for *in vitro* PP5 activity assay (Swingle *et al*., 2007). Together, our results indicate that PP5 could act as an upstream phosphatase for AMPK and inhibition of PP5 by palbociclib subsequently leads to AMPK activation, autophagy induction, and apoptosis.

Similar to that observed from pharmacological inhibition of PP5 by palbociclib, knockdown of PP5 led to upregulation of p‐AMPK (Fig. S4A) and impaired cell growth (Fig. S4B). Moreover, palbociclib treatment contributed to stronger signals of apoptosis and AMPK phosphorylation in PP5‐silenced cells (Fig. S4C). These results further support the concept that PP5 is one of the molecules that mediate the cytotoxic activity of palbociclib.

### 3.4 PP5/AMPK axis, but not CDK4/6, determines the efficacy of palbociclib in HCC cells

**Figure 4:**
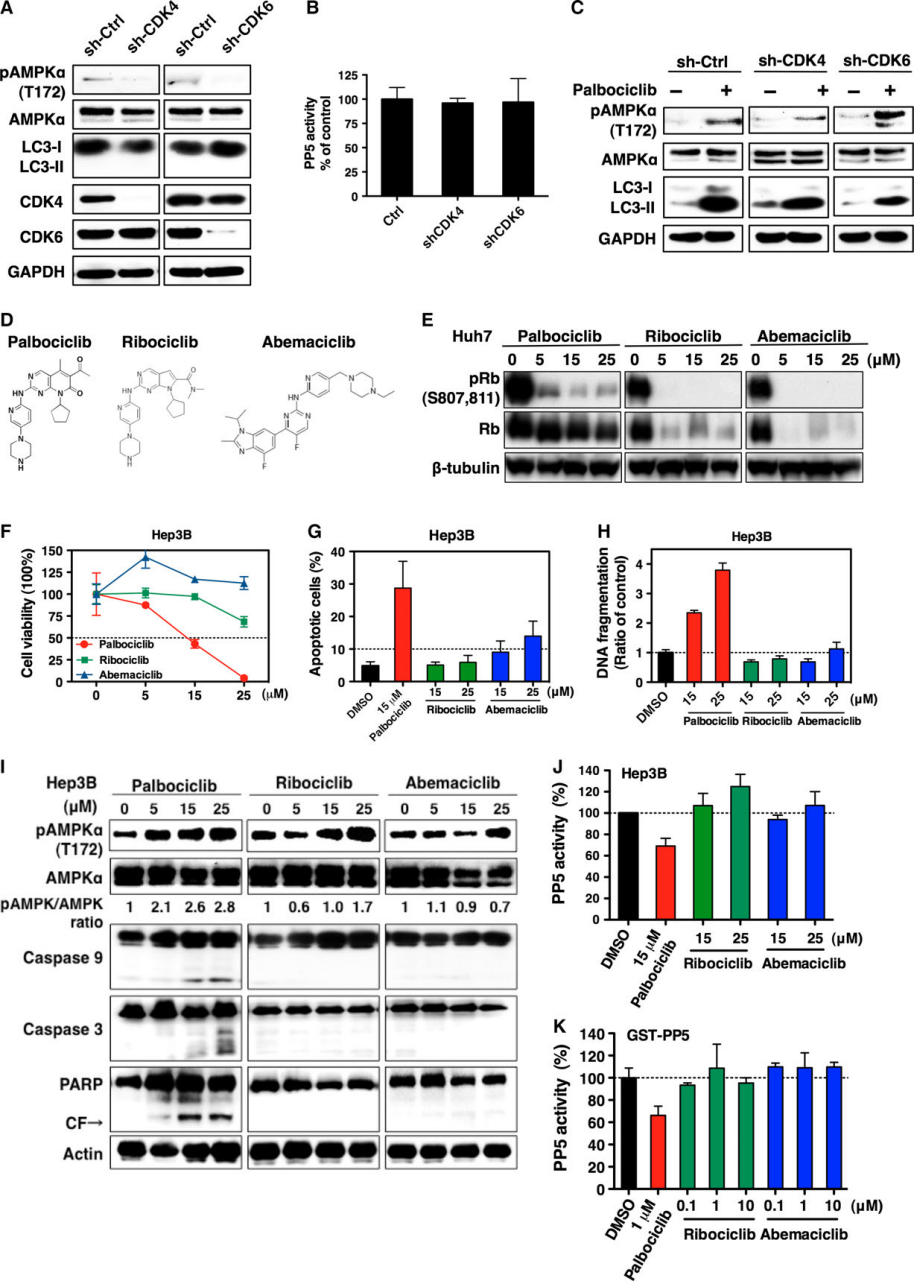
Palbociclib exhibits anti‐HCC activities in a CDK4/6‐independent manner. CDK4 or CDK6 in Hep3B cells was knocked down by lentivirus‐based RNA interference. Lysates of CDK4/6‐knockdown Hep3B cells were subjected to immunoblotting in (A) and PP5 activity measurement in (B). (C) Palbociclib induces AMPK phosphorylation and autophagy in CDK4/6‐knockdown Hep3B cells. The stable CDK4/6‐knockdown cells were incubated with palbociclib (15 μm) for 24 h and analyzed by western blotting. (D) Structures of CDK4/6 inhibitors. (E) CDK4/6 inhibitors decrease phosphorylation of Rb. Huh7 cells were treated with different doses of CDK4/6 inhibitors for 24 h. The amount of Rb phosphorylation was determined by western blotting. (F–H) Effects of CDK4/6 inhibitors on cell viability, apoptosis, and DNA fragmentation. (I) Effects of CDK4/6 inhibitors on AMPK phosphorylation and apoptosis‐related signals. After 24 h of drug treatment, the cells were subjected to western blot analysis. AMPK phosphorylation level was quantified by the ratio of band intensities of phospho‐AMPKα vs. AMPKα. (J) Effects of CDK4/6 inhibitors on PP5 phosphatase activity in Hep3B cells. (K) Effects of CDK4/6 inhibitors on GST‐PP5.

It was not clear whether palbociclib targets the PP5/AMPK axis via CDK4/6. To tackle this question, we used two different strategies to inhibit CDK4/6 activities. First, CDK4/CDK6‐knockdown Hep3B cell lines were generated by transducing lentiviruses encoding shRNA against CDK4, CDK6, or a scrambled control. The successful knockdown of CDK4 or CDK6 was confirmed by immunoblotting (Fig. 4A). Interestingly, CDK4/6 knockdown in Hep3B cells did not lead to AMPK phosphorylation, LC3‐II upregulation, and PP5 activity inhibition as palbociclib did (Fig. 4A,B). Moreover, palbociclib induced AMPK activation and autophagy in both control and CDK4/6 knockdown Hep3B cells, suggesting that palbociclib elicits its anti‐HCC activity independently of the CDK4/6‐Rb pathway (Fig. 4C).

Second, we evaluated the anti‐HCC effects of two other CDK4/6 inhibitors, ribociclib (LEE011; Novartis, Basel, Switzerland) and abemaciclib (LY‐2835219; Eli Lilly, Indianapolis, IN, USA), which have recently reached early clinical trials (Geoffrey Shapiro *et al*., 2013; Jeffrey Infante *et al*., 2014). The structures are shown in Fig. 4D. As potent CDK4/6 kinase inhibitors, all of them effectively inhibited Rb phosphorylation at comparable doses in Huh7 cells (Fig. 4E). Among the three CDK4/6 inhibitors, only palbociclib showed potent cytotoxic effects in Hep3B cells, as evidenced by MTT, sub‐G1, and DNA fragmentation assay (Fig. 4F–H). Consistently, cleaved PARP and caspase 9 were shown in palbociclib, but not in ribociclib‐ or abemaciclib‐treated cells (Fig. 4I). Furthermore, PP5 activity and AMPK signals were examined in drug‐treated Hep3B cells (Figs 4I–J and S5A). Ribociclib and abemaciclib did not suppress cellular PP5 activity, nor did they stimulate AMPK signals in Hep3B cells. Ribociclib at high dose slightly increased AMPK phosphorylation, but failed to clearly exhibit cytotoxicity in Hep3B cells (Fig. 4I, middle panel; also see Fig. 4G–H). Cotreatment of ribociclib and metformin, a known AMPK activator, further enhanced AMPK phosphorylation and induced cell death. On the other hand, ribociclib or metformin alone did not (Fig. S5B). The results suggest that AMPK activation is a critical determinant for cellular sensitivity to CDK4/6 inhibitors. GST‐PP5 activities were not affected by ribociclib and abemaciclib, either (Fig. 4K). Together, our results demonstrate that palbociclib exerts anti‐HCC activities by targeting the PP5/AMPK axis beyond CDK4/6.

### 3.5 *In vivo* anti‐HCC activity of Palbociclib

**Figure 5:**
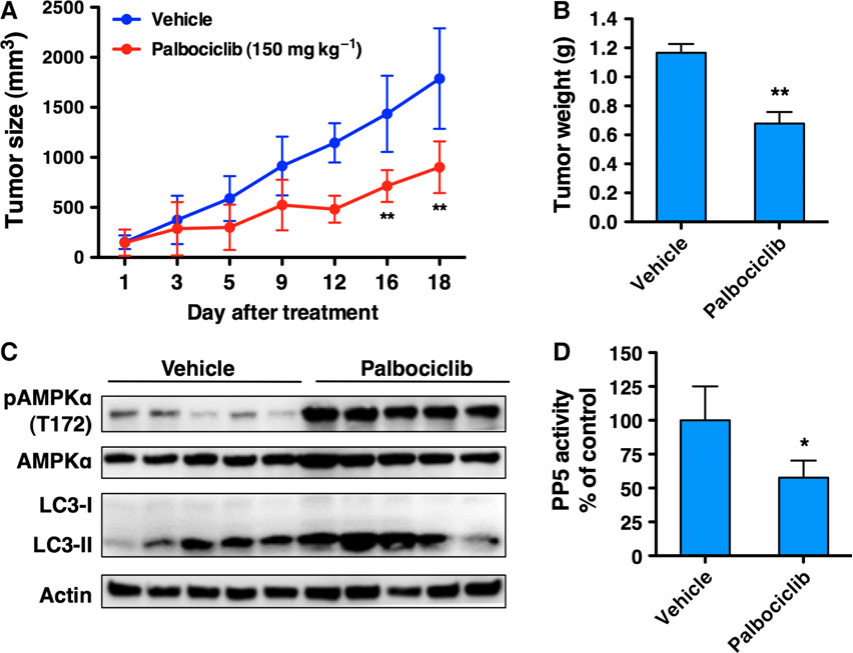
*In vivo* effects of palbociclib in Huh7 xenograft nude mice. (A,B) Palbociclib suppresses Huh7 xenograft tumor growth and weight. Nude mice were subcutaneously implanted with 5 × 10^6^ Huh7 cells. The mice received vehicle or palbociclib (150 mg·kg^−1^) orally every three days. The tumor area was measured twice a week. At the end of treatment, the tumors were harvested and the tumor weights were determined before lysis. Points/bars, mean (*n* = 5); bars, SD. **P <* *0.01. (C) Western blot analysis of pAMPK, AMPK, and LC3 in Huh7 tumors. (D) PP5 activity in Huh7 tumors. *P <* *0.05.

Palbociclib potently affects the induction of autophagy and cell death through PP5/AMPK axis *in vitro*. To determine the antitumor activity and mechanisms of palbociclib *in vivo*, nude mice bearing Huh7 xenografts were used. As shown in Fig. 5A,B, palbociclib significantly inhibited tumor growth and reduced tumor weight in Huh7 xenograft mice. The mice in the control group maintained body weight throughout the treatment, while the mice that received 150 mg·kg^−1^ palbociclib showed a slight loss in body weight (Fig. S6). To explore the molecular mechanisms underlying the tumor‐suppressive effect of palbociclib, the xenografted tumors were subjected to PP5 activity assay and western blot analysis. As with the mechanisms identified *in vitro*, there were higher levels of phospho‐AMPK and LC‐3 II, and reduced PP5 activity in tumors treated with palbociclib (Fig. 5C,D). In summary, these results demonstrate that the antitumor activity of palbociclib is highly associated with its regulation of the PP5/AMPK pathway.

### 3.6 Expression of PP5 is a novel biomarker associated with aggressive clinical features

**Figure 6:**
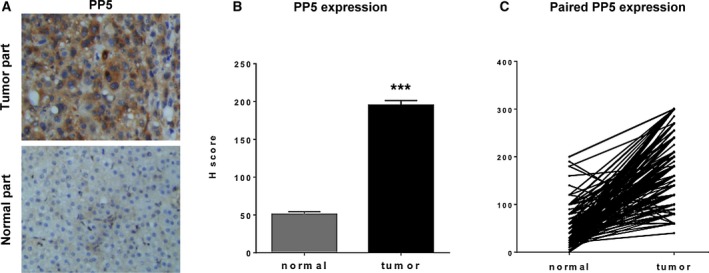
Expression of PP5 in human HCC tissues. (A) Expression of PP5 in HCC tumors and normal liver tissue. The protein expressions of PP5 in the HCC tumors and its adjacent normal part were analyzed in 153 patients with HCC by IHC. Representative images were shown here. (B) PP5 expression in HCC tumors is significantly higher in HCC tumors than in normal liver tissue. The expressions of PP5 were quantified by *H*‐score and compared by Student's *t‐*test. *P *<* *0.001. Bar, mean; error bar, SE. ***P <* *0.001. (C) PP5 expression in paired tumor and normal liver tissue. Each dot represented the *H*‐score obtained from indicated tissues, and each line linked an individual patient.

To understand the clinical relevance of PP5 activity in HCC, we analyzed the protein expression of PP5 in the tumor tissues obtained from 153 patients with HCC (Table S1). As shown in Fig. 6A,B, the expression of PP5 was significantly higher in the tumor part than in the normal liver tissue (*P *<* *0.001). Moreover, when we compared the expression of PP5 in the tumor to its paired normal part, most of the patients had increased PP5 expression in the tumor tissues (Fig. 6C). Notably, the average serum level of alpha fetal protein in patients with high PP5‐expressed tumors was significantly higher than in patients with low PP5‐expressed tumors (*P* = 0.037) (Table S2). It has been shown that pAMPK is frequently downregulated in HCC tissues (Zheng *et al*., 2013). Consistently, we found that pAMPK expression is relatively low in HCC tumors associated with differential PP5 levels (Fig. S7).

## 4 Discussion

Targeting the cell cycle as anticancer therapy has been actively investigated for decades, and recently, tremendous progress has been made as the highly selective CDK4/6 inhibitor palbociclib received FDA approval for breast cancer treatment. To date, palbociclib has been applied in multiple preclinical models, but its use for HCC therapy remains elusive. Here, we showed the kinase‐independent anti‐HCC activity and the associated molecular mechanism of palbociclib that may be clinically relevant. Additionally, we are the first to report the therapeutic potential of the PP5/AMPK axis for HCC‐targeted therapy.

Although previous studies have indicated that RB levels determine the sensitivity to CDK4/6 inhibition (Dean *et al*., 2010; Konecny *et al*., 2011; Logan *et al*., 2013; Michaud *et al*., 2010), we and others have observed some activity of palbociclib that is independent of RB levels in HCC cells. First, palbociclib induced significant apoptosis in RB‐deficient Hep3B cells (Fig. 1). Rivadeneira *et al*. (2010) reported that palbociclib consistently suppresses tumor growth under Rb‐deficient conditions. These results suggest that the RB dependence is not general for CDK4/6 inhibitor treatment. In the context of HCC, other CDK4/6 substrates or unidentified novel targets may bypass the drug effects. A recent report showed that palbociclib, but not ribociclib, modulates the autophagy pathway and AKT signaling in squamous cell lung carcinoma (Sumi *et al*., 2015), which correlates with the autophagy induction by palbociclib in HCC found in this report. Moreover, high levels of palbociclib (7.5 and 15 μm) have the potential to target c‐Jun/COX2 signaling to affect breast cancer metastasis (Qin *et al*., 2015). Our results, together with these reports, suggest that palbociclib has additional targets that may contribute to its antitumor activities and impact clinical practice.

Conversely, we found that palbociclib possesses additional activity to induce HCC cell apoptosis compared to the other high‐specificity CDK4/6 inhibitors, ribociclib and abemaciclib (Fig. 4). These results also indicate that palbociclib exerts its cytotoxic effects in addition to canonical CDK4/6‐Rb signaling. Currently, palbociclib and ribociclib are undergoing phase II trials for advanced HCC patients and chemoembolization in patients with locally advanced HCC, respectively (NCT01356628 and NCT02524119). Our findings will provide useful information for ongoing and future clinical studies.

Interestingly, a recent report shows that palbociclib induced a reversible cell cycle arrest, which is dependent on RB, in human liver cancer cell lines (Bollard *et al*., 2016). By analyzing cell cycle alteration in response to palbociclib treatment, Bollard *et al*. demonstrated the on‐target effect of palbociclib at low doses (< 1 μm for *in vitro* experiments). Herein, we found that palbociclib could induce HCC cell death in the micromolar range. Even though the high dose of palbociclib used in the present study does not necessarily fall within a clinically relevant range (Schwartz *et al*., 2011), a previous report showed that the mean concentrations of palbociclib in murine tumor samples at 6 h postoral administration at 100 mg·kg^−1^ could be up to 25 163 ng·g^−1^ (~ 52 μm) (Nguyen *et al*., 2010). It appears that palbociclib may be able to achieve high drug exposure in tumor regions. Thus, the clinical need clearly exists to examine the effect of high‐dose palbociclib treatment and to unveil the molecular mechanism behind. In the current study, we have demonstrated that the PP5/AMPK axis determines the efficacy of palbociclib treatment in HCC. *In vitro* and *in vivo* experiments showed that p‐AMPK levels were tightly associated with the anti‐HCC activity of palbociclib. Moreover, blockade of AMPK signalings by chemical inhibitors or siRNA targeting AMPK attenuated palbociclib‐induced cell death. The findings described herein suggest that AMPK activation represents a critical molecular event for palbociclib‐induced apoptosis in HCC cells. It would be rewarding to further investigate whether AMPK could serve as a biomarker for predicting clinical response to palbociclib.

Here, we showed that Hep3B and PLC5 cells displayed differential sensitivity to palbociclib. So far, the underlying mechanism responsible for intrinsic resistance of PLC5 remains largely unknown. Our results showed that knockdown of PP5 overcomes the resistance to palbociclib in PLC5 cells (Fig. S8), indicating that the abundance of endogenous PP5 plays a role in the resistance. Additionally, our results showed that palbociclib did not suppress cellular PP5 activity from PLC5 cell lysates (Fig. 3C). These results suggest that there might be unidentified factors that protect PP5 from palbociclib inhibition in PLC5 cells. Several physiological activators of PP5 have been identified, such as AA, long‐chain fatty acid‐CoA esters, and S100 proteins (Ramsey and Chinkers, 2002; Yamaguchi *et al*., 2012). S100 protein family is the largest subgroup of calcium‐binding proteins and has recently been implicated in tumorigenesis (Bresnick *et al*., 2015; Donato, 2001). As intracellular calcium sensors and extracellular factors, S100 proteins participate in a wide range of cellular activities, including calcium homeostasis, cell proliferation, cell motility, cell invasion, and motility (Chen *et al*., 2014). To date, at least 25 members in this family have been described. We speculate that the abundance of the PP5 physiological activators, such as S100 proteins, might be higher in the resistant PLC5 cells than in other sensitive cell lines, thereby leading to palbociclib resistance. However, the association of S100 proteins with HCC remains unclear. A few studies have analyzed the expression of S100 proteins in HCC (Cui *et al*., 2006; Hua *et al*., 2011; Kim *et al*., 2009). Further investigation is required to examine the proposed mechanism.

This is the first report to link PP5 phosphatase and AMPK. AMPK is a well‐studied metabolic tumor suppressor (Luo *et al*., 2010). The molecular mechanism by which PP5 modulates AMPK activation remains unclear. AMPK phosphorylation at Thr172 can be negatively regulated by Ser/Thr protein phosphatases such as PP2A, PP2C, PP1, and PPM1E (Davies *et al*., 1995; Garcia‐Haro *et al*., 2010; Voss *et al*., 2011; Wu *et al*., 2007). Of note, the phosphatase‐binding toxins (i.e., okadaic acid), which have been commonly used in earlier studies to assess the action of PP1 and PP2A, have a similar inhibitory effect toward PP5 (Swingle *et al*., 2007). Therefore, the involvement of protein phosphatases in AMPK regulation using PPase toxins should be carefully reinterpreted. Moreover, the components within the AMPK complex also contribute to AMPK phosphorylation. AMPK exists as a heterotrimeric complex composed of a catalytic α subunit, a scaffold β subunit, and a regulatory γ subunit. Myristoylation on β subunit and the binding of AMP/ADP to γ subunit have been shown to facilitate AMPK activation (Oakhill *et al*., 2010, 2011). Further studies on the molecular interplay between PP5, AMPK complex, and the upstream kinases/phosphatases will uncover the detailed mechanisms and benefit future targeted drug development.

The aberrant expression of PP5 in HCC tissues (Fig. 6) suggests that PP5 plays a role in the development of HCC. So far, the molecular mechanism by which PP5 contributes to HCC tumorigenesis is not clear. It has been reported that ablation of PP5 by shRNA inhibits HCC cell growth (Feng *et al*., 2015). Our results showed that inhibition of PP5 by palbociclib leads to HCC cell death. These findings indicate that aberrant PP5 expression may aid the progression of HCC and suggest that PP5 is a potential novel target for anti‐HCC therapy.

In conclusion, taken together our current findings show that palbociclib possesses potent anti‐HCC activity by inducing both autophagy and apoptosis via the PP5/AMPK signaling pathway. In prior studies, the CDK4/6‐RB pathway was asserted to determine the sensitivity of CDK4/6 inhibitors. Here, we revealed a novel kinase‐independent action of palbociclib on AMPK activation and PP5 inhibition. Furthermore, we showed the clinical relevance of PP5 in HCC. According to our preclinical investigation, we believe that palbociclib treatment and targeting PP5/AMPK axis have great potential for HCC therapy.

## Author contributions

KFC and CWS conceived the idea, and KFC supervised the manuscript. FSH and YLC designed and performed the *in vitro* experiments. FSH, YLC, and MHH analyzed the data and wrote the manuscript. PYC and YLC provided clinical samples and performed the tumor tissue analysis. MHT, LJC, YJH, CTS, MJC, and TIC performed the validation and animal experiments. All authors reviewed this manuscript.

## Data Accessibility

## Supporting information


**Fig. S1.** Caspase activation in HCC cells after palbociclib treatment.
**Fig. S2.** Palbociclib induces autophagy in HCC cells.
**Fig. S3.** Palbociclib induces phosphorylation of ASK1 and JNK in a concentration‐dependent manner.
**Fig. S4.** Effect of PP5 knockdown on AMPK and cell growth.
**Fig. S5.** Effect of CDK4/6 inhibitors on AMPK phosphorylation.
**Fig. S6.** The body weight of mice received vehicle or palbociclib (150 mg·kg^−1^).
**Fig. S7.** The representative immunohistochemical patterns of PP5 and pAMPK in clinical HCC samples.
**Fig. S8.** Knockdown of PP5 overcomes the resistance to palbociclib in PLC5 cells.
**Table S1.** Characteristics of the study cohort (*n* = 153).
**Table S2.** Characteristics of HCC patients with high and low PP5 expression.
