# Supplementary material for: Palbociclib induces activation of AMPK and inhibits hepatocellular carcinoma in a CDK4/6‐independent manner
Source: Mol Oncol. 2017 May 15;11(8):1035–49. doi: 10.1002/1878-0261.12072 (PMC5537702; doi:10.1002/1878-0261.12072)
Supplement: Supplementary file 1 — Fig. S1. Caspase activation in HCC cells after palbociclib treatment. Fig. S2. Palbociclib induces autophagy in HCC cells. Fig. S3. Palbociclib induces phosphorylation of ASK1 and JNK in a concentration‐dependent manner. Fig. S4. Effect of PP5 knockdown on AMPK and cell growth. Fig. S5. Effect of CDK4/6 inhibitors on AMPK phosphorylation. Fig. S6. The body weight of mice received vehicle or palbociclib (150 mg·kg−1). Fig. S7. The representative immunohistochemical patterns of PP5 and pAMPK in clinical HCC samples. Fig. S8. Knockdown of PP5 overcomes the resistance to palbociclib in PLC5 cells. Table S1. Characteristics of the study cohort (n = 153). Table S2. Characteristics of HCC patients with high and low PP5 expression. [file MOL2-11-1035-s001.pdf]

Palbociclib induces activation of AMPK and inhibits hepatocellular carcinoma in a CDK4/6 independent manner

Feng-Shu Hsieh, Yao-Li Chen, Man-Hsin Hung, Pei-Yi Chu, Ming-Hsien Tsai, Li-Ju Chen, Yung-Jen Hsiao, Chih-Ting Shih, Mao-Ju Chang, Tzu-I Chao, Chung-Wai Shiau, Kuen-Feng Chen

Table of contents

Supplementary Methods……………………………………………………………2

Supplementary Fig. S1..4

Supplementary Fig. S2..6

Supplementary Fig. S3..7

Supplementary Fig. S4..8

Supplementary Fig. S5..9

Supplementary Fig. S6..10

Supplementary Fig. S7..11

Supplementary Fig. S8..12

Supplementary Table S1……………………………………………………………13

Supplementary Table S2……………………………………………………………14

Supplementary Methods

Immunofluorescence and acridine orange staining

1×10^5 HCC cells were seeded on coverslips 1 day before drug treatment. Immunofluorescence staining to detect active caspase-3 was performed with Alexa Fluor® 488 conjugated cleaved caspase-3 (Asp175) antibody (Cell Signaling Technology, no. 9669; diluted 1:100). Briefly, cells were washed once with PBS, and fixed in freshly prepared 4% formaldehyde for 20 min. After permeabilization with PBS containing 0.3% Triton X-100 for 5 min, cells were blocked in 5% BSA-PBS for 1 hour. After blocking, cells were incubated with Alexa Fluor® 488 conjugated cleaved caspase-3 (Asp175) antibody overnight and then mounted with DAPI. For acridine orange staining, drug-treated cells were stained with 5 μg/ml acridine orange (Sigma-Aldrich, St. Louis, MO; A8097) in serum-free medium at 37°C for 30 min. Cells were washed twice with PBS, fixed in 4% paraformaldehyde, and washed with PBST (0.05% Tween 20 in PBS) before they are mounted. Samples were observed under a Zeiss Imager A1 upright microscope. Positive acidic vacuoles or lysosomes were stained as red foci in the cytoplasm while DNA bound-acridine orange was detected as a green signal.

Caspase 3/7 activity assay

1×10^4 HCC cells were seeded on chamber slides (ibidi μ-slide 8 well, no. 80826) 1 day before drug treatment. The cells were exposed to various concentrations of indicated drug for 16 hours. Caspase-3/-7 activity was measured with the CellEvent™ caspase-3/-7 Green Detection Reagent (Invitrogen, Cat. No. C10723). Cells were incubated with 5 μM CellEvent™ caspase-3/-7 green detection reagent in PBS/5%FBS for 30min at 37°C in the dark. Stained cells were observed under Leica DM IRE 2 Inverted fluorescence microscopy.

Lentiviral infection of HCC cells

Recombinant lentiviruses were used for knockdown cell line establishment. pLKO.1-puro vector expressing non-targeting (NT) control shRNA (pLKO TRC025) or shRNAs targeting PP5 (TRCN0000002801 & TRCN0000002802) were used. Prepared lentiviral supernatant was added to the HCC cells at a multiplicity of infection (MOI) of 2. After infection for 24 hours, the virus media were replaced with normal culture media supplemented with 10% FBS and 1% PenStrep, and incubated for another 24 hours. Then the media were replaced with the one containing 2-4 μg/ml puromycin. The infected cells were assayed after at least 72 hours puromycin selection. The short hairpin RNA (shRNA) reagents and the recombinant lentiviruses were obtained from the National Core Facility for Manipulation of Gene Function by RNAi, Academia Sinica, Taiwan.

(Related to Fig.1)

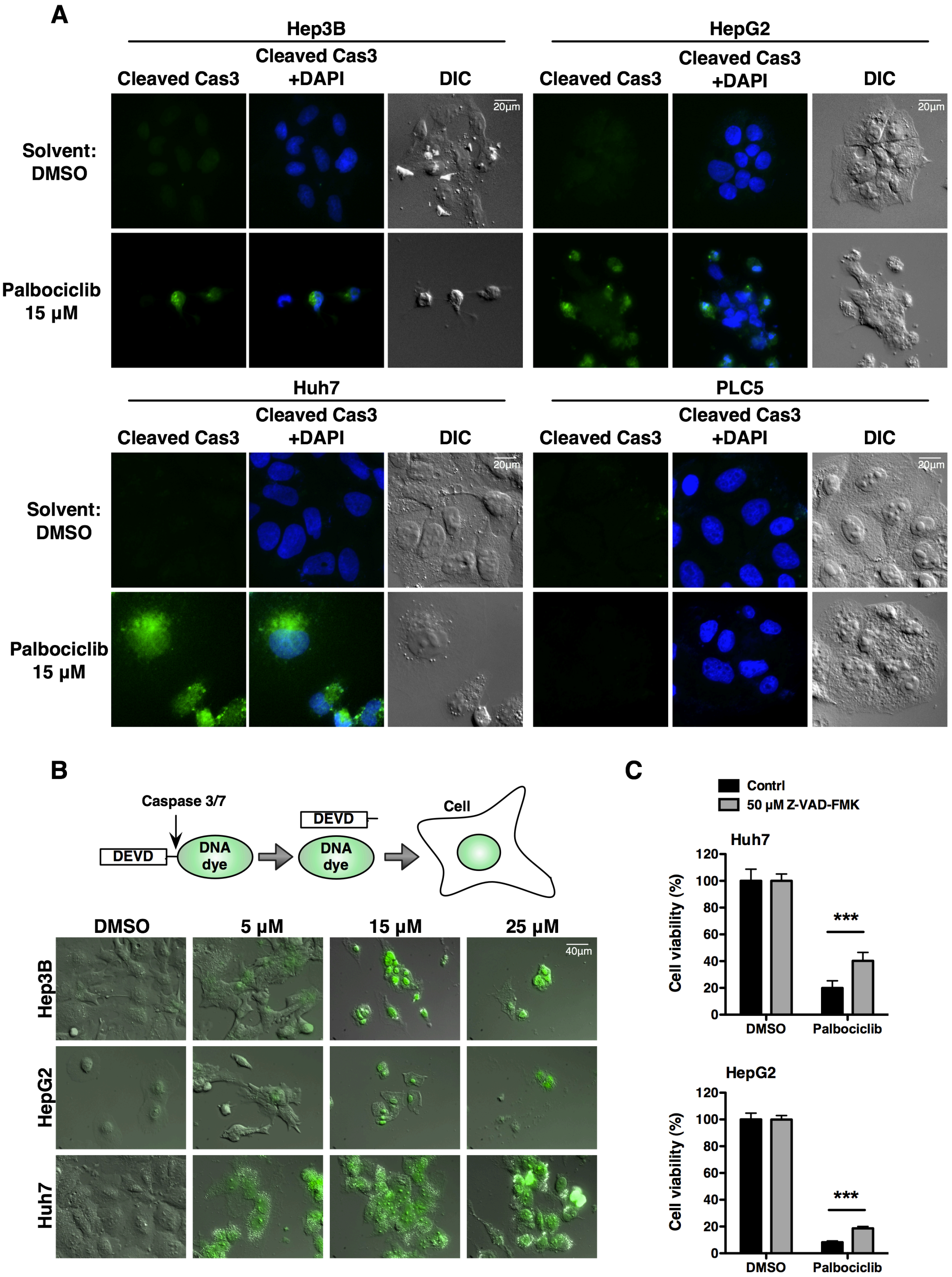

Supplementary Fig. S1. Caspase activation in HCC cells after palbociclib treatment. (A) Cleaved Caspase 3 immunofluorescence. HCC cells were treated

with palbociclib (15 μM) for 24 hours. (**B**) Top, a schematic representation for CellEvent™ caspase-3/-7 Green Detection Reagent. Once the substrate is cleaved by activated caspase-3/-7, the probe becomes fluorescent and free to bind to DNA. Bottom, Caspase-3/-7 activation in palbociclib-treated HCC cells was observed using fluorescence microscopy. Fluorescence images merged with DIC images are shown. **(C)** HCC cells were pre-treated with Z-VAD-FMK (50 μM) for 2 hours, followed by palbociclib (25 μM) treatment, cells were further cultured for 24 hours before cell viability assay was performed. ***, $P < 0.001$

(Related to Fig. 1)

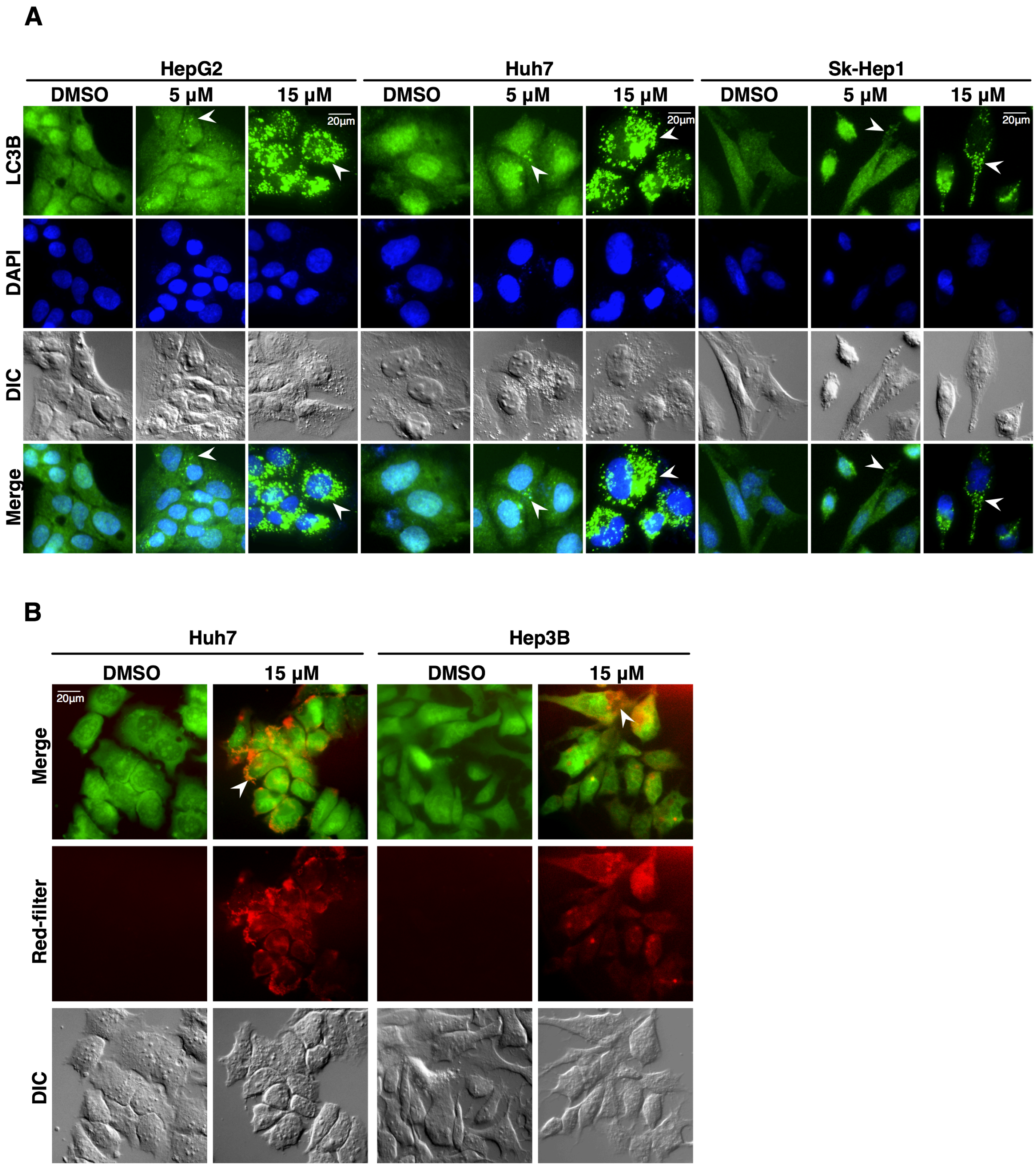

Supplementary Fig. S2. Palbociclib induces autophagy in HCC cells. (A) LC3B immunofluorescence in palbociclib-treated HCC cells (HepG2, Huh7, and Sk-Hep1 cells). Arrowheads indicate LC3 positive autophagosomes. Nuclei were counterstained with DAPI (blue). Scale bar, 20 µm. **(B)** Acridine orange stain of palbociclib-treated HCC cells (Huh7 and Hep3B). Arrowheads indicate acidic vesicular organelles (Red staining). The corresponding DIC (Differential interference contrast) images were shown below.

(Related to Fig. 3)

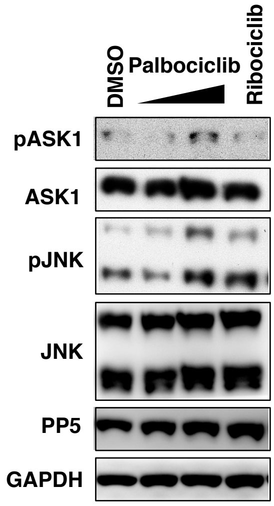

Supplementary Fig. S3. Palbociclib induces phosphorylation of ASK1 and JNK in a concentration-dependent manner. Hep3B cells were exposed to palbociclib (5 or 15 μM) for 24 hours. DMSO and Ribociclib (15 μM) treatment served as negative controls. Western blot was performed with indicated antibodies.

(Related to Fig. 3)

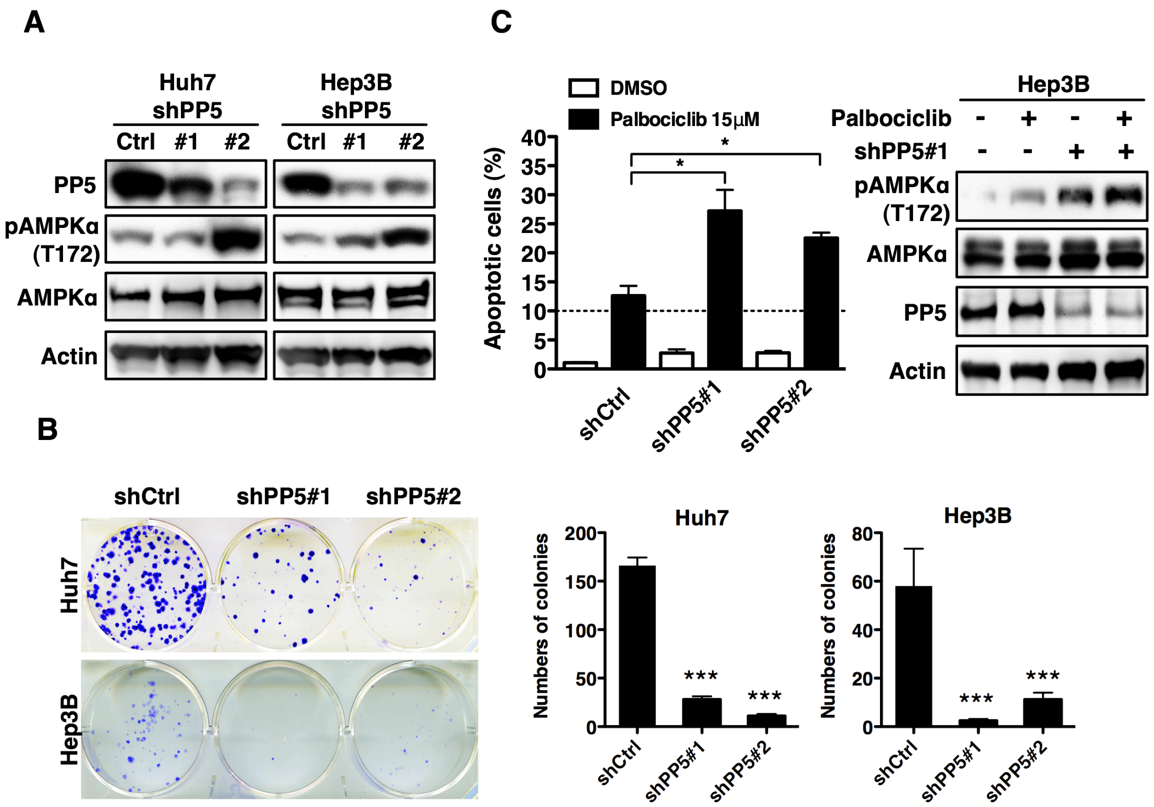

Supplementary Fig. S4. Effect of PP5 knockdown on AMPK and cell growth. **(A)** Western blot analysis of PP5, pAMPK, and AMPK in PP5 knockdown HCC cells. **(B)** Knockdown of PP5 decreased the colony formation of HCC cells. *Right,* statistical analysis of colony numbers in HCC cells. **(C)** Knockdown of PP5 increased palbociclib-induced apoptosis and phosphorylation of AMPK.

(Related to Fig. 4I)

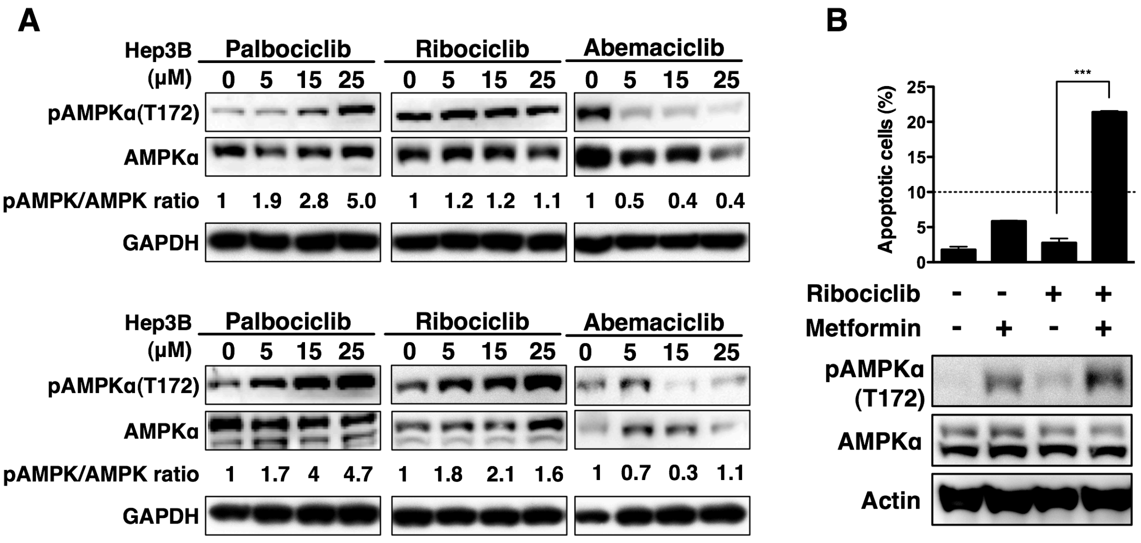

Supplementary Fig. S5. (A) Effect of CDK4/6 inhibitors on AMPK phosphorylation. After 24 hours of drug treatment, the cells were subjected to western blot analysis. AMPK phosphorylation level was quantified by the ratio of band intensities of phospho-AMPKα versus AMPKα. (B) Co-treatment of metformin and ribociclib induced cell death in Hep3B cells. Cells were exposed to ribociclib at 25 μM and/or metformin at 10 mM for 72 h. Apoptotic cells were determined by flow cytometry.

(Related to Fig. 5A)

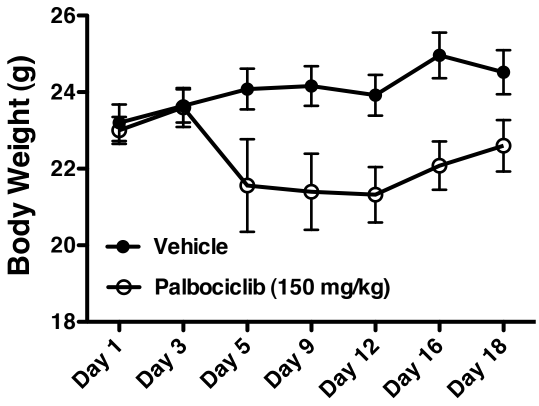

Supplementary Fig. S6. The body weight of mice received vehicle or palbociclib (150 mg/kg).

(Related to Fig. 6)

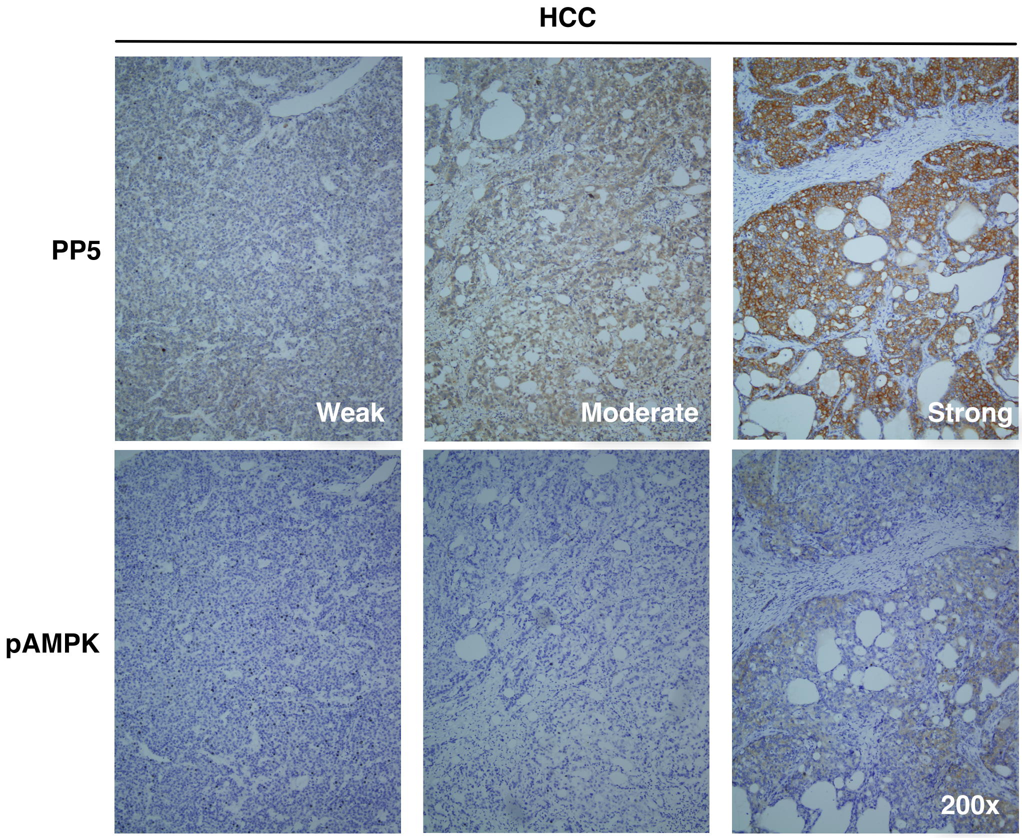

Supplementary Fig. S7. The representive immunohistochemical patterns of PP5 and pAMPK in clinincal HCC samples.

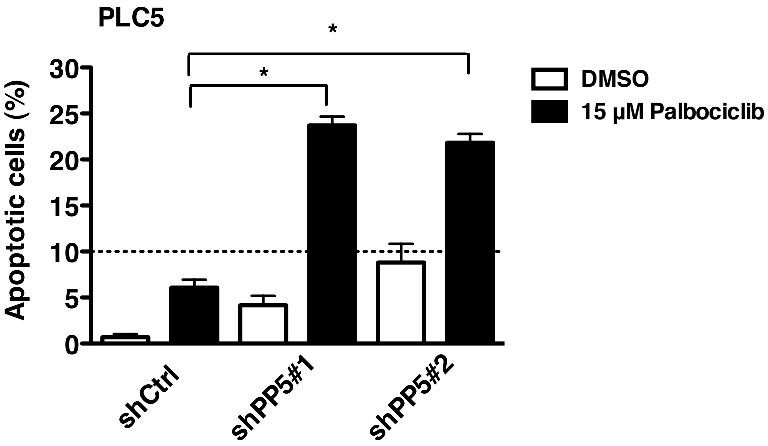

Supplementary Fig. S8. Knockdown of PP5 overcomes the resistance to palbociclib in PLC5 cells. Cells were exposed to 15-μM palbociclib for 24 h. The apoptotic cells were analyzed by flow cytometry.

Supplementary Table S1. Characteristics of the study cohort (n = 153)

Characteristics	Number	%
Age [median (IQR)]	64 (57-72)	
Male sex	111	72.5
TJCC stage		
1	49	32.0
2	64	41.8
3	17	11.1
4	2	1.3
Missing	21	13.7
Tumor differentiation		
1	5	3.3
2	70	45.8
3	71	46.4
Missing	7	4.6
HBV-associated	65	42.5
HCV-associated	44	28.8

Supplementary Table S2. Characteristics of HCC patients with high and low PP5 expression

Characteristics	Low PP5 (n = 95)	High PP5 (n = 58)	*p*
Poor tumor differentiation	35 (36.8%)	36 (62.1%)	0.055
Serum AFP (mean)	536	4017	0.037
HBV infection	38	27	0.914
HCV infection	23	21	0.611
